# Status and Perspectives of Commercial Aircraft Morphing

**DOI:** 10.3390/biomimetics7010011

**Published:** 2022-01-07

**Authors:** Michelangelo Giuliani, Ignazio Dimino, Salvatore Ameduri, Rosario Pecora, Antonio Concilio

**Affiliations:** 1 REDAM, Research and Development in Applied Mechanics, 83046 Avellino, Italy; info@redam.it; 2 The Italian Aerospace Research Centre (CIRA), Department of Adaptive Structures, 81043 Capua, Italy; i.dimino@cira.it (I.D.); s.ameduri@cira.it (S.A.); 3 Industrial Engineering Department, University of Naples “Federico II”, 80125 Napoli, Italy; rosario.pecora@unina.it

**Keywords:** morphing wings, adaptive structures, control systems, embedded kinematics, distributed actuator and sensor networks

## Abstract

In a previous paper, the authors dealt with the current showstoppers that inhibit commercial applicability of morphing systems. In this work, the authors express a critical vision of the current status of the proposed architectures and the needs that should be accomplished to make them viable for installation onboard of commercial aircraft. The distinction is essential because military and civil issues and necessities are very different, and both the solutions and difficulties to be overcome are widely diverse. Yet, still remaining in the civil segment, there can be other differences, depending on the size of the aircraft, from large jets to commuters or general aviation, which are classifiable in tourism, acrobatic, ultralight, and so on, each with their own peculiarities. Therefore, the paper aims to trace a common technology denominator, if possible, and envisage a future perspective of actual applications.

## 1. Introduction

Morphing wings are an excellent means to increase aircraft performance, as demonstrated by many researches in the field. Indeed, the bibliography is rich in studies, attainments, and tests of devices implemented on significant mock-ups. Furthermore, the history of aviation itself counts many trials for installing variable-shape wings on the aircraft at different extents. However, as a critical classification of the accessible instruments and tools is tried, it is necessary to differentiate technology by size. In fact, one of the most relevant concepts in the field regards the proper scalability of the proposed architectures. Yet, this basic idea is not entirely assessed, in spite of some recent specialized publications on the matter. Because aerodynamics, mechanics, structural dynamics, aeroelasticity, and so on depend on different physical laws, in turn involving a large number of diverse parameters, any result attained on a given configuration is not easily exportable to others. A direct consequence of this statement is that what is tested on models or a specific aircraft is not generally transferrable to full-size systems or different classes of planes. This fact implies huge complications for the design process. Therefore, as commercial aircraft is targeted, a filter shall be applied to cut or better evaluate many examples appearing in literature, no matter how relevant and fascinating. In that sense, and with that conceptual limitation, with the ultimate aim of addressing actual applicability of morphing technology on commercial aircraft, the survey herein illuminated some recent attainments and recalls on the several kinds and sizes of airplanes. One of the most important factors that limits the actual development of the morphing technology is related to the necessity of performing a careful assessment of its impact on the aircraft in terms of benefits and drawbacks. This step is not trivial, and in turn, implies a precise definition of the vehicle mission or missions, where the targeted device is supposed to be employed, and a perspective of the consequence of the modified configuration on the whole operational envelop. Weight penalties, effects on safety inspections, and so on could determine the viability of a specific innovative system, and should always be evaluated versus the expected advantages. For instance, if an additional component could favor and simplify the take-off phase, it shall be demonstrated it does not negatively affect the other phases of the flight, neither the planned maintenance nor other aspects of the regulation requirements. For instance, within the MAS project, funded by DARPA in the USA, NextGen Aeronautics developed an adaptive wing capable of largely modifying its aspect ratio [1]. The target was achieved by a truss system and a deformable skin that stretched and shrunk the wing to assume loitering or diving configurations, for instance. Another major example is given by the aircraft model recently developed and flown by NASA, where an adaptive winglet was used to change wingspan or assume positive or negative cant angles, depending on the flight necessities [2]. Finally, some years ago, NASA also exposed a futuristic architecture where elementary devices were combined together in order to provide an almost-continuous form variation to the hosting vehicle. Such a digital wing, as it was called, then evolved in a complete aircraft, all made of such simple subunits that were named voxel, perhaps recalling pixels in a usual digital image [3,4]. A similar concept was also developed as a part of a multi-morph robotic system [5].

There are many examples concerning manned aircraft-size; indeed, such proposals run all over the flight history, starting from the 1910s and 1920s [6,7]. In the first case [6], a patent was issued concerning kinematic systems made for modifying the external shape of the front and aft parts of the wing, while the second example [7] proposed a configuration able to self-adapt the profile of bi- and triplanes. A significant step was accomplished as the F111 was equipped with a wing capable of continuously varying its trailing and leading-edge cambers. The aircraft made a huge test campaign, the results of which are still a milestone in the sector of the adaptive aeronautic structures [8,9]. The improved capability in loitering, diving, and maneuvering was clearly assessed, finally demonstrating the potential of such an envisaged technology. It was only ten years later that a study was presented to illustrate the applicability of that concept to large-size commercial aircraft, along the wake of the development of the Airbus planes [10]. Finally, large-size prototypes were assessed between 2015 and 2017, with two large projects in Europe and the USA. The EU SARISTU project designed, manufactured, and tested a full-size wing section in wind tunnel, demonstrating the feasibility of realizing an adaptive wing for commercial aircraft applications [11]. It integrated three different morphing systems on a 5.5-m-span demonstrator, positioned at the leading and trailing edges, and at the winglet, respectively. A NASA/AFRL joint project (Adaptive Compliant Trailing Edge, ACTE), involving Gulfstream and Flexsys, designed and tested a compliant adaptive flap prototype in flight, aimed at replacing all the conventional control surfaces on the wing [12]. Experiments were carried out in 2014 and gave full demonstration of the capability and potentiality of the envisaged technology. Many other studies have been carried out since then, such as [13]. In Canada, significant activities on morphing applications on commercial aircraft have been carried out in recent years. The CRIAQ MDO 505 project focused on developing methodologies for designing adaptive aerodynamic components (winglet and aileron) of a full-scale regional aircraft prototype [14]. Other studies at the Research Laboratory in Active Controls, Avionics, and Aeroservoelasticity Laboratory (LARCASE) at the École de Technologie Supérieure (ÉTS) did concentrate on the development of morphing winglet and horizontal tail systems for the CRJ-700 aircraft [15,16,17], and the Cessna Citation X business aircraft [18,19]. Currently, AG2, a large European project within the Clean Sky 2 Green Regional Aircraft program, aims at the full-scale, functional flight testing of an adaptive winglet in 2022 [20]. This list is not exhaustive at all; a devoted paper is necessary to deal and discuss the recent developments of morphing wing technology, but the cited examples are enough to understand how far the research has evolved, in the authors’ opinion.

Coming to full manned aircraft, both civil and military, it may be of a certain interest to recall actual flying systems, flown onboard of very famous planes, such as the Concorde (droop fuselage nose) or the F-14 (variable sweep angle) [21,22]. Other existing static systems are the aircraft current stored on a typical air carrier that needs to fold their wings, or tilt rotor vehicles that are used to orientate differently their rotors (and the hosting lift-generating supports) as a function of the flight phases. For the sake of completeness, it should also be considered that morphing devices, even if very far away from a smart structure architecture, have always been employed on airplanes, as flaps, slats, ailerons, and rudder are necessary to fly. No aircraft could face even the most elementary flight phases demand without adaptive capabilities. Withstanding the remarkable knowledge developed over the years on deformable wing shapes, and the consolidated technology that allows even complicated systems to fly while respecting the demanding regulations, it is almost natural to wonder why such an idea has not yet being implemented on real aircraft.

It is about 20 years that a revolutionary morphing wing concept was introduced by Northrop-Grumman within the DARPA-sponsored Adaptive Wing project, (1997–2002), where a completely embedded and continuous system was developed and tested, even if in the limited environment of a wind tunnel and mounted on a UCAV [23]. It is important to ask what showstoppers have slowed down the technology development, and how far engineering is from the actual implementation of that vision. As it often happens, there is not a unique answer. Instead, there is a concurrency of reasons that span from the purely technical point of view to the need to properly accomplish the existing regulations while keeping low operational costs and high safety levels. It may be that an important item, such as aeroelasticity, enters in one or more of the listed reasons; however, it is preferred to deal with it separately since it may represent an issue usually neglected, while it should be instead duly taken in account. New, breakthrough technologies led to new aircraft configurations and new design approaches. As a consequence, it shall be expected that new methodologies and design processes are needed to better tackle the new outline of the vehicle system and its mutated complexity. However, assessed procedures required time, and it is almost physiological that a time shift occurs between the rising of a new technology and the set-up of adequate design means. This fact inevitably leads to a limited estimation of the complete effects of the innovation on the comprehensive performance, affecting the vehicle’s assessment along its whole lifecycle. On the other hand, it should be recognized that the future perspective of a more and more electric aircraft could be a facilitator of morphing on the aircraft of the next generation. The wide presence of cabling and connections will make it easier to design an extensively broad network of actuators and sensors, which are supposed to be the core part of the system. Cabling design, the choice of a distributed or centralized power supply architecture, and the influence of the induced magnetic fields represent some of the key aspects of the electric craft. The further presence of morphing systems could be one of the key aspects to consider before coming to a final choice.

In this paper, the authors try to list the weak points of the chain and how they can be overcome to guarantee the installation of morphing systems onboard of commercial aircraft. The identification of the class of vehicles is essential for the abovementioned scalability issues, but even for the very different requirements and expectations that the various airplane classes could arise. The variety itself of the difficulties makes it necessary to articulate such a survey properly. Technical aspects will be faced first, design issues will follow, and finally, operational and implementation aspects will conclude the analysis. This paper is not meant to be an exhaustive vision on morphing technology, which instead would certainly require volumes and volumes. It simply aims at moving some questions on the latest developments and, hopefully, stimulate a different focus on topics sometimes neglected.

## 2. Preliminary Considerations

Before going into detail, some fundamental reflections shall be carried out to properly introduce the following concepts.

There is no special concern of the existing regulations about the use of morphing systems. Even the most revolutionary of those ones is based on accessible and well-assessed technology, or clear ideas. It is instead the exasperation of the proposed concepts and the novelty according to which they are arranged together that raises the challenge. For instance, the most popular architectures, which de facto divide the scientific and technology community in two parts, are based on kinematic systems and compliant structures. Both of them are largely present on commercial aircraft, even if at a smaller extent, than it would be needed for a fully morphing system.

As of now, there are two major architectures including any kind of morphing solutions: kinematics, such as the one proposed in SARISTU, or compliant, as well as the one proposed in ACTE. In spite of an accurate analysis showing the large similarity of the two concepts, with some confined strong exceptions (such as the design of the load-bearing frame and the realization of the lability elements, allowing the movement), the discussion concerning their limitations and perspectives would be very different. Therefore, the authors decided to focus on kinematic systems herein, shifting a larger dissertation to a next work, which presumably will take advantage of the conclusions that will be drawn here.

## 3. Technical Aspects

**Figure 1:**
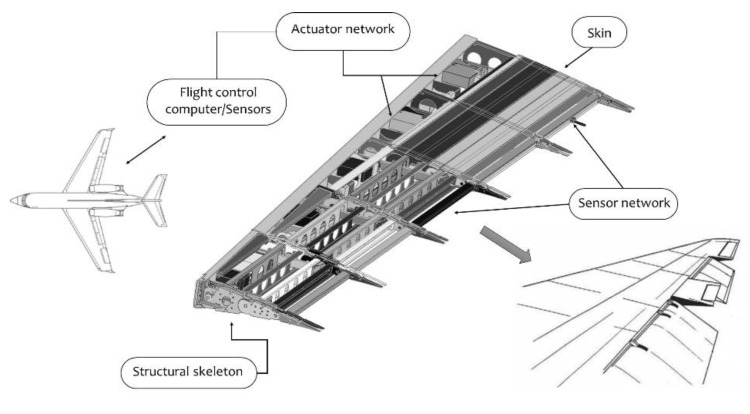
Schematics of the morphing wing device subsystems.

The core of any morphing system is composed of a structural skeleton, sensor and actuator networks, a control system, and an enveloping skin (as schematically shown in Figure 1). Following, the single elements will be recalled, and their major weakness points will be highlighted. Such topics will be finally summarized in a table for an immediate understanding and transferring of the result of this analysis.

### 3.1. Structural Skeleton

The structural skeleton is the part of the system enabling the change in shape and concurs to withstand of loads related to all the configurations that it can be morphed into. Therefore, its design needs to combine adequate compliance to efficiently accommodate large shape changes and enough stiffness to counteract external loads with proper margins of safety.

Three different structural solutions may be adopted to comply with this requirement.

- *Articulated mechanism*: the skeleton is composed of structural elements interconnected by hinges and leverages and arranged into a mechanism with one or more degrees of freedom. As a mechanism, the skeleton cannot withstand external loads if its degrees of freedom are active.
  In this case, the actuation chain plays a crucial role as it works along the active degrees of freedom to change the shape of the skeleton and adsorbs part of the external loads while constraining its movements.
- *Compliant structure*: the structure is a rational assembly of subcomponents with no active degrees of freedom. The actuation chain induces elastic deformations of one or more subcomponents to morph the skeleton according to the desired shape. In this case, the actuators have a less significant role in withstanding the external loads as the skeleton has an intrinsic (and tailored) stiffness. However, this does not mean reduced demand for actuation power since the actuators must counteract the stiffness of the structure to morph its shape.
- *Hybrid structure*: combination of the two structural solutions listed above.

Irrespective of the adopted structural solution, the skeleton cannot be seen as a critical path, as long as it is concerned with standard parts and the usual way of assembly. This is even more true if the articulated mechanisms are addressed. Of course, it should be noted that the number of elements may increase significantly with the number of shapes the skeleton can assume. Such a number is associated to the number of degrees of freedom that the system needs to be given as a function of the targeted performance of the adaptive architecture. Connected to that, the number of interfaces grows as well, and that fact further increases complexity. This aspect also involves the number of connections and, therefore, the joints associated with a non-neglectable boost of weight.

### 3.2. Sensors

As for the structure, sensor networks are not majorly critical, in principle. There are many certified devices in commerce, suitable for aircraft applications (civil or military); therefore, the access to that technology is not a matter of concern. However, again, linked to the number of degrees of freedom that the structure wants to be given, the number of necessary sensors also grows. This fact has at least two major impacts: the number of cables may explode, involving room and weight necessity; and the number of devices may significantly expand, luckily with no direct impact on room and weight penalties. Both of those issues give rise, however, to an increase in architectural complexity.

### 3.3. Actuators

Again, a distributed degree-of-freedoms system does necessarily require a certain number of actuators to work properly. The alternative is to reduce the number of motors but increasing the mechanical system ramifications to distribute unequally and in a controlled way, the actuator action. In that case, the number of active devices would anyway increase, shifting the needs of adequately commanding a certain line to mechanical switch, in turn consisting of micro-actuators and a further increased complexity of the system architecture, with increased cabling either.

Since a distributed system cannot be based on hydraulic or pneumatic actuators for reasons of accessible room and weight, the use of electromechanical actuators (EMA) is almost mandatory. Even though this kind of device is studied in many research projects worldwide, many aspects regarding reliability and certification are still open. In fact, in order to realize a proper morphing system, an actuator shall be small enough to fit the structural body while guaranteeing the necessary level of forces. A certain number of devices is accessible on the market, but many of them suffer export limitations so that technology cannot be marked as “mature”. Furthermore, because many of them are developed for the military UAV market, some civil certification requirements could not be satisfied.

A significant issue concerning the actuators is the need to take them as a part of the structural system. In fact, because the skeleton shall allow lability in order to be moved, it follows that the actuator shall bear part of the load (if it is removed, the system is a mechanism with unconstrained degrees of freedom). Therefore, the actuator shall usually concur to sustain external and internal loads. Since the aircraft is an isolated system, the force absorbed by the actuator is transferred to the structure through the connection interfaces. The connection of the motors to the structure (usually the main spar, with the attachment deployed on its web) shall be re-designed to consider that unusual load.

### 3.4. Control System

Conformally to the structural skeleton and the sensor network, control systems are widely used on all types of aircraft, civil and military, manned and unmanned, models or real-size. Therefore, technology accessibility is not an issue. However, what it does change with morphing systems is the number of variables to be controlled, linked again to the targeted DOF. The number of variables may affect the control algorithm complexity, the control system stability, and, as a consequence, certification. The process may terminate in a reduction in the operational envelop, and the resulting architecture could be heavily resized as a function of the imposed constraints.

In the case of control systems, the requested functions also have an impact on it. Many operations could require a complex analysis of the internal and external variables, while ensuring the whole machine is stable and works properly. It may be argued that robotics have dealt with and partially solved this kind of problem for a long time; the easy answer is that the environmental conditions are very different from a quasi-static, ground analysis.

### 3.5. Skin

This is perhaps the most critical element in the set-up of an adaptive wing. It is also the sole element, among the cited ones, whose criticality is not driven from the degrees of freedom. It has to behave in a certain way independently of how many movements shall be realized. Instead, it depends on the extension of the envisaged deployment.

The key of a morphing skin functionality is its capability of being extended for deformations that can attain 10% with a minimum amount of forces while guaranteeing high transversal stiffness. The first property follows the necessity of limiting the effort required to the actuators, while the second one derives from the need to preserve geometrical continuity under the action of aerodynamics that could otherwise generate irregularity in the wing outline. Since the most obvious solution to the excess of deformability is the increase om thickness, weight and accessible volume is consequently affected, with severe impacts on the overall system economy. Unfortunately, if not well addressed, this augmented stiffness also causes a degradation of the performance in the other directions, leading to added loads to the actuator system.

### 3.6. General Issues

All mentioned subsystems (structure, sensors, actuators, logic, and skins) present an additional problem: the problematic scalability, here intended as the transportability of the technology from a class of vehicle to another one. This aspect cannot be always addressed just by scaling the subsystems, since their performance does not follow the geometrical scaling factor. To cite some example, hydraulic actuators suited for large-scale applications cannot be miniaturized over a certain level and must be substituted by more compact actuators. A structure conceived to change the chamber of a wing of a small UAV cannot be resized to fit larger classes of vehicles since its flexibility would be dramatically altered and too rigid, and a system originally compliant could be converted into a kinematic chain. For this reason, designers are often forced to dramatically change the structure layout, the type of sensors and actuators, and the physical principle at their basis.

The summary of weaknesses and crititcalities associated to the different components of a morphing system is synthetically reported in Table 1.

## 4. Design Issues

### 4.1. Approach

Even though the adaptive structures herein referred are an evolutionary concept of the existing systems, it is undoubtful that there are significant impacts on the design process. In fact, the skeleton is made no more as a single block with a certain shape whose changes under the aerodynamic and internal load could be eventually taken into consideration. Here, we have a bundle of structures that should be separately verified under the presence of the prescribed excitations. For instance, if there is a droop nose whose geometry may change between 0 and 20 deg (inclination of the mean with respect to the nominal chord), the system should be verified tested for different configurations. This aspect arises a first issue: how many steps shall be considered? (1°, 2°, 5° Or even less?) The answer severely impacts the operation to assess the established design.

Even if this topic was rarely considered till now, the necessity of referring to a “zero” shape shall be considered. This issue has not been emphasized until now because the larger part of the studies refers to existing aircraft, whose capability has been augmented by the insertion of adaptive parts. Therefore, the basic geometry was intrinsically defined. What will be the impact of imagining a morphable aircraft with variable shape along with its life and its mission. What will be the choice of the designer? While it may be easy for commercial aircraft to indicate the cruise condition as the reference one, this item may be dramatic for military or UAV platforms.

An immediate consequence of this consideration concerns the specification’s issue. How will the customer or the design staff (aiming, for instance, at placing a novel aircraft on the market, with new and interesting features) measure the envisaged requirements? In this case, the lack of tradition and consolidated process plays an important role. Perhaps the most critical aspect is that, without assessed indications, there is the risk of an indefinite time for producing the final schematic and several iterations with the specs generator before a shared vision is set up.

Another important aspect is represented by the highly integrated level of the different components of a morphing system. This aspect, crucial for the correct functioning, has a dramatic impact on the design approach. With specific reference to the structure of a drop nose system, it is ideally possible to distinguish between the main subparts, as the internal structure that transmits deformation from the actuator to the skin, and the skin itself, which must assure a specific shape and distribute the external load to the interior parts. It is evident that, despite this distinction, it is not possible to face the design of the components separately. To further extend this reasoning, we can imagine that these components are made of different materials, for instance a multilayer hybrid skin (outer part of soft elastomeric material, inner part of honeycomb), connected to an interior supporting structure, made of a super-elastic alloy. The design approach must rely either upon a unique multiphysics tool or a shell open source software, which can handle different specific tools.

Still, with reference to the high integration level, structures actuated through shape memory alloys (SMA) active parts present a further complication: the pre-load. Since the exploitability of a SMA actuator is strictly related to the martensite phase concentration, in turn produced by the pre-load level, the surrounding structure shall withstand the conventional loads and also produce an adequate stress level within the SMA. This evidently leads the structure to work in an unconventional way, characterized by a generally higher stress level.

### 4.2. Tools

Something that runs in parallel with the design approach is related to the available tools. Usually, they refer to structural systems, while recently many proprietary tools are developed to consider the aerodynamic loads to assess the target wing shape. In fact, since the aerodynamic characteristics of a body are a consequence of its shape, the deformed configuration is essential to guarantee the envisaged performances. This non-trivial approach complicates a lot as new DOFs are added, whose activation depends on the number of kinematic architectures and electromechanical actuation systems. The targeted simulator shall not only consider the presence of such complex systems, but it has to correctly simulate the presence of the structural elements allowing displacements (hinges, gears, and so on).

Some researchers have recently demonstrated the impact of rotational and translational constraints on the simulated structural response, which can drastically affect the final result. In this context, it is no more sufficient to have FE codes at disposal, combined with aerodynamics and enriched with multi-body capabilities. The detailed simulation of particulars is requested. The easy criticism to this statement is that, after all, the prospected kinematics, is not that different by the current flaps and ailerons, for instance. The answer is yes, of course. What it does change however, is the number of these components spread all over the aircraft. Instead, since the presence of many discontinuities linked to the distributed kinematics arises, it is expected that the number of singularities will explode in the analysis, in turn requesting deeper investigations. Again, this impacts the time and costs of the design process.

Complementarily, the use of tools for issuing appropriate load conditions is affected. Again, a bundle of geometries shall be considered, together with many local imperfections. Either, since the structure is interrupted by many discontinuities, the usual way to provide the internal solicitation characteristic may be affected by some forced assumption that should be necessarily removed to avoid excessive conservativisms.

### 4.3. Aeroelasticity

This part of the design is treated separately for its sound peculiarity. It refers to the analysis of the interactions between inertial, elastic, and aerodynamic forces arising on the structure when it moves in the airstream. If inertial forces are neglected, the static behavior of the system can be investigated by referring to the previously outlined concepts, concerning tools, and approaches. In the case of relevant inertial forces (high speed/accelerations), the dynamic stability of the system becomes a crucial design aspect, and flutter analysis is required since the preliminary design stage of the adaptive structure. It is already expected that the system, with augmented DOFs, will have a more complex aeroelastic response in terms of modes involved in potentially unstable coupling mechanisms. This is closely linked to the characteristics of the many systems composing the morphing aircraft as the combination of structural parts, kinematic architectures, and actuation devices. Their integrated response deserves great attention from the modeling point of view. Since the system is generally characterized by largely distributed characteristics, its dynamic condensation cannot follow the standard approaches, and the resulting output shall necessarily be made of a considerable number of grids, elements, and properties. Considering that the aeroelastic evaluation shall be carried out on the complete aircraft, this situation naturally leads to complex models, whose analysis is not trivial and presumably deserves many iterations to be finally assessed.

Once more, the main question is concerned with the discretization that shall be implemented to ensure a correct outcome. In other words, given the geometrical domain that represents the possible configurations the morphing aircraft may assume, what is the step that can be considered sufficient to explore it? There is not a standard answer yet or a shared criterion for defining it. A practical approach would be to adopt specific solutions to overcome potential instabilities detected along with the study. The installation of balancing masses is among the most effective solution to fix aeroelastic issues with minimal impact on the consolidated design of the item. Nevertheless, because different configurations shall be explored, there is no guarantee that a practical solution working for a given configuration is also effective for the others. The problem is, again, associated with the introduction of suitable tools that can consider any possible issue for any possible configuration before, and not after, it is detected.

### 4.4. Coupling

A rarely treated topic concerns the cross interactions of the different morphing systems that can be deployed over the wing or the whole aircraft in general. In fact, since the aircraft is an isolated system, the stresses and strains generated by the action of a single adaptive device transmit along the whole structure and may give rise to unwanted or at least unpredicted deformations. It may be perhaps assumed, with a certain level of confidence, that a morphing rudder cannot induce aileron deformations, to a certain extent, but this hypothesis cannot be held anymore if different wing components are referred to as flaps and slats. This phenomenon has been reported in the literature and simply means that a morphing aircraft cannot be, at least in principle, designed by parts, but should be approached as a whole, since the first time. Again, the associated difficulty is linked to the available tools and the lack of a significant background experience in dealing with this kind of problem extensively. It is worth mentioning that many hints may be taken from the movables currently being mounted on a generic aircraft, but the larger density of adaptive systems would require different attention on the specific thematic.

### 4.5. Preexisting Subsystems

When developing a morphing system, generally two alternative scenarios appear to the designers: either the aircraft is completely new and the general layout can be defined ex-novo as the morphing system, or the aircraft already exists, its architecture is well-consolidated, and the morphing devices must replace some conventional subsystem.

Generally, this second option is the most common, and the designers must meet requirements strictly related to the preexisting layout. Especially in this case, the impact of the novel morphing system plays a critical role. Needed energy, structural connections, and working modality must be accurately weighted to minimize the impact of the novel system onto the rest of the aircraft and avoid any undesired penalization due to the overlapping of the pre-installed technologies.

To cite an example, for the specific case of an adaptive chamber flap or a drop nose, the designers will face the problem of ideally removing the conventional subsystems and related actuation layouts and replace them with the novel architectures, whose connection to the wing box and whose allocation of the actuation subsystems are really different and, especially for compliant mechanisms, implies a specific load transmission path to the wing box. The total weight (here intended as the sum of the morphing systems themselves and the wing structure) from one side cannot be compared to the weight of the replaced conventional devices since they do not include the kinematic chain and the actuation systems; however, from the other side, they must be limited as much as possible to not undermine the benefit expected by the new technologies.

Another example is represented by a morphing leading edge’s impact on conventional preinstalled ice protection systems. These systems (hydraulic, pneumatic, electromechanics, and thermal) are conceived to act onto rigid structures, and parts of them are supported by the interior structure and the skin. If the surrounding structure morphs, the problem of the stability from one side, and of the effectiveness from the other side, arises. In this case, the introduction of a morphing device cannot be faced separately from the integration of other subsystems or from the attempt of including some additional functionality (ice removal) in the morphing device itself.

The summary of weaknesses and crititcalities associated to the design process of a morphing system is synthetically reported in Table 2.

## 5. Manufacturing and Assembly

### 5.1. Manufacturing

Manufacturing represents one of the most challenging aspects of the development path of a morphing system. The possible use of unconventional materials, such as hybrid metallic-ceramic components and smart materials, may further complicate the scenario. Such peculiar components may require specific processes, as the sintering of piezoelectric powders [24], or suited melting and milling processes for shape memory alloys [25]. Topology and chemistry of variable stiffness parts may be very complex, and can be hardly compatible with conventional manufacturing processes (milling, melting, and bonding). The coexistence of more materials, each with their own characteristics, implies engineering concurrency and a multidisciplinary approach.

The most relevant concern is associated with the number of parts, which can be very relevant. In fact, the architectures of the morphing device and the hosting structure require necessarily to suit each applicable section, resulting in different elements at the various positions, directly affecting the layout of the kinematic systems. A rationalization of the subsystems outline is usually necessary, including actuator and sensor networks, with potential impacts on the effectiveness of the guidance logic.

Since the production process needs a maturation involving setting and optimization of the concurrent parameters and the demonstration of the reproducibility within certain levels, many questions arise, related to the need of a maturation period for the related industrial processes. 

### 5.2. Assembly

The assembly process of an integrated morphing system is generally different and more complex than conventional ones, featuring the embedding of the whole components with the aim of producing clean surfaces. Even though, from a traditional point of view, it is still convenient to distinguish among logic, sensors, actuators, structure, and skin, system efficiency is strongly related to their amalgamation level. This directly impacts the design process, of course, as anticipated in the previous section, and even has an impressive influence on the assembly process.

To cite some examples, a morphing structure may require a certain level of pre-load, needed to assure the correct working condition of SMA actuators if present [26]. This, in turn, requires dedicated processes and devoted rigs, jigs, and tools for imposing the envisaged stress, while putting together the multitude of system components. Another example regards the installation of sensor networks that become an intimate part of the structural components, with the associated cabling and electronics. Their interface may be critical for the correct functioning of the whole system [27]. The same applies for actuation systems that, in this case, also play an important role as load-bearing elements, with relevant consequences on the structural integration.

Increased DOFs imply the multiplication of interfaces; however, multi-component architectures may be hard to handle and require specific tools, and non-homogeneous material composition must be carefully treated during handling and fixing operations to avoid inappropriate solicitations and fracture onset. Other topics that may arise relate to possible junctions of adaptive structures with classical materials, such as SMA, in composite materials [28] or piezo transducers within elastic components [29].

Strictly related to morphing system efficiency, assembly processes shall mitigate undesired free-play and gaps within kinematics. In this wake, the most peculiar aspect of adaptive structures with respect to classical systems shall be cited, based on their intrinsic nature. The increased DOF and the necessary lability until the actuation system is integrated and blocked make the structure movable during the integration. There is no more a static configuration that grows rigidly step by step, but is instead the creation of a chain with multiple capabilities of movement. Apart from the clear general difficulty, the need of dedicated jigs and tools is evident, to limit the displacement at the most even during the most common processes for standard structures (drilling, riveting, bolting, and so on). This simple consideration may have tremendous consequences on the production time and costs.

The presence of uncommon materials, even for apparently usual components, such as the skin, should not be neglected. The necessity of allowing large strains with minimal stress penalties, which would directly impact the actuation system performance, naturally brings attention towards elastomeric solutions, with all its limitations. These materials, however, have critical issues with respect to traditional assembly methods. Elastomers are hard to be drilled, and related parts may be hard to be joined by riveting.

A last consideration regards the current scenario. The current industrial trends aim to simplify building procedures for minimizing times and costs. The introduction of a morphing system is certainly conflictual with those tendencies.

The summary of weaknesses and crititcalities associated to the manufacturing and assembly process of a morphing system is synthetically reported in Table 3.

### 5.3. Ground Testing

The ground testing of the assembled morphing component is a fundamental task that is generally required before its final integration into the aircraft, especially when dealing with prototypal architectures.

The primary purpose of the tests is to prove that the structure can safely withstand the most severe loads expected in service (*static tests*) and that the system is morphable in compliance with the required morphed shapes (*functionality tests*). Resonance tests can also be carried out for some specific components to validate the dynamic models developed during the design phase.

Unlike conventional structures, morphing systems demand the tests of different configurations along with several sets of loads. 

Therefore, pre-test simulations are highly recommended to rationally define a limited number of test conditions enveloping the most relevant combinations of configurations and loads. Once the enveloping conditions are determined, the following general procedure can be followed for each condition:

(a) *Functionality test*: the test article is morphed and unmorphed repeatedly by activating the actuators through the control system. During this process, the external shapes of the test article are acquired (by 3D scan or displacements’ measurements at a set of control points) and compared with target ones. The outcomes of this comparison provide a means of evaluation for the reliability and the robustness of the system’s functionality.
(b) *Static test*: The test is carried out to prove that the structural system is able to withstand loads without detrimental deformation or failure. A careful installation of the whiffle tree has to be carried out to apply operative loads without constraining the natural degrees of freedom of the morphing system; for instance, in the case of the segmented articulated ribs (as shown in Figure 1), segmented load saddles must be used to not prevent the relative rotation of rib blocks. The natural degrees of freedom of the structure must be constrained only by the actuators (in power-on mode) and related transmission lines; the entire actuation chain is, therefore, a relevant part of the test article and must be sensorized to get info on load-induced strain and elastic displacements. In addition, the power adsorbed by the actuators to maintain the shape of the loaded structure must be measured to ensure that the actuators still possess enough authority to morph the system in operative conditions.
(c) *Functionality check*: after unloading the test article, the functionality tests are repeated to check that the stresses and strain fields arise during the static test and that the level of power adsorbed by the actuators to counteract the external loads have not compromised the morphing performances of the system.

## 6. Operations

### 6.1. Maintenance and Repair

Maintenance and repair operations have a significant impact on running costs and thus on market attractiveness as well as flight safety [30]. A morphing system disregarding this aspect risks nullifying the benefits produced in terms of performance and environmental impact.

The time needed by maintenance operation is the most critical aspect since it directly influences the usage time of the aircraft. Thus, attention is paid to the effectiveness of the procedures and on the reliability of the interventions, as well as to their compliance with the current regulations.

A morphing system poses different issues, strictly related to the nonconventional architectures often hardly to inspect, the presence of non-conventional materials with not enough studies supporting life cycle and reliability, sensorial architectures non-conventional themselves and also subjected to new logic of monitoring, and sophisticated logics of control suited for multi-distributed DOF systems. However, more than anything, the deep level of integration of a morphing system is critical from a maintenance point of view [31].

When introducing a new concept onboard the aircraft, the impact on maintenance procedures and operations is one of the most relevant pieces of information that should be provided in order to understand if, and to what extent, the benefits of the technology are real. A dedicated analysis is then necessary. A maximum allowable time period shall be established before a maintenance action is required for announced failures. Latent failure will be managed by a scheduled maintenance task. If this approach is taken, component mean time between failures (MTBF) is the basis for establishing the check interval time, e.g., time intervals between maintenance and operational checks or inspections.

Coherently with the basic hypotheses of this work, kinematic systems and electromechanical motors are considered. Sensor networks are not so different from the usual ones, and control system softwares follows classical rules. Where available, a service history of similar or same components in a similar or the same environment should be used. Impact of new materials shall be duly considered.

As a basic consideration, it can be said that a maintenance plan can be prepared on the basis of the existing experience on the different items. However, some differences arise. Generally speaking, the integration of the components makes it hard to deal with them separately; this aspect has an important fall-out on the repair process.

Repair operations are, in practice, affected by the lack of knowledge on the new materials involved, which determine uncertainty on the type and effectiveness of the repair action. Damage on monolithic or quasi monolithic structures must be adequately handled, from one side, to avoid the substitution of the entire component, but must be also properly secured in compliance to the specific unconventional working modality of the part. Furthermore, repairing a distributed sensorial system may require invasive operations, often non-localized on specific parts, but involving the entire network.

Starting from motors, even with the assumption of having excellent life expectations, the simple fact to have a large number of such devices imposes to reduce the inspection times by a figure inversely proportional to the implemented systems. The same applies to kinematic architectures and related components. Since these elements are commonly used and treated, no special attention shall be paid to them.

Embedding sensors in structural components generates some issues. Apart from the obvious difficulty of detecting the faulting device in a large and distributed array, the presence of malfunctioning in an integrated net led to the necessity of dismissing the whole segment. In turn, this fact generates the need for a suitable design of the interfaces connecting the different channels, so that cabling shall not be revolutionized by such a substitution. Furthermore, if the system has the goal of providing critical information on system faults, it should be designed to maximize its maintainability by allowing, for instance, quick, easy, and safe access for repair or replacement or by preventing inappropriate connections or interpretations. 

While structural parts and software dedicated to system guidance are definitely standard parts whose maintenance procedures are well coded and assessed, something different concerns the skin, if elastomeric-based solutions are addressed. This kind of material is particularly sensible to the external conditions and suffer the action of the environmental agents, and is not reparable. Definitely, this aspect could be overcome by novel attainments in the material sector, but that is the situation right now. So, damage on such surfaces, essential to preserve the geometry, can lead to the substitution of the entire segment.

The presence of a large number of parts, all different the ones from the other, brings added complexity to the problem. A suitable repair process shall necessarily rely on a number of accessible spare parts, with significant impact on stock sizes and cost. These reflections translate into specs for the design that shall duly consider the interfaces, the access to the different elements, and the issues for repair and substitutions. It is almost obvious that a complete re-styling of the procedures is useful for the augmented complexity of the system, in spite of many elements are still traditional.

The challenges of maintenance of morphing structures could be summarized in just a couple of questions, as follows. How can the lack of knowledge on new materials, systems, and configurations be handled to obtain a final product competitive also from the maintenance point of view? How can regulation update fill the gap with industrial applications?

### 6.2. Safety

To comply with the EASA CS-25 requirements considering both operational implications and crew work load, a morphing wing device, as any other equipment and systems installed onboard aircraft, shall be designed and installed so that any catastrophic failure is extremely improbable and does not result from a single event. The entire system or the individual subsystems must be substantiated by analysis and tests in airplane or in a mock-up installation to determine proper performance and prevent failures. This is generally guaranteed by adequate design avoiding stress concentration, instability, and corrosion; a dedicated logic of control to prevent dangerous conditions and mitigate the load distribution; and a monitoring system able to sense the current status of the architecture. All these aspects are, however, very challenging: designing a structure flexible and rigid at the same time means to manage the stress and load distribution beyond conventional schemes; logics of control shall be able to handle more DOFs than usual and must exhibit higher performance and a higher rate of affordability; and health monitoring systems shall be suited for the early detection and quantification of the damage, and supported by a logic aimed at mitigating the related effects.

Novel aircraft functions associated with adaptive systems impose the introduction of new fault trees for catching the associated risks at the level of the complete airplane. Before conducting a detailed safety assessment, a functional hazard assessment (FHA) of the airplane shall be prepared by considering the potential failures of airplane level functions due to the system’s malfunctions. This phase is concerned with the operational vulnerabilities of the system rather than with a detailed analysis of the actual implementation.

An inverse relationship is commonly accepted between the average probability of fault occurrence per flight hour and the severity of its effects. Catastrophic failures must be extremely improbable and must not result from a single failure; extremely improbable failure conditions are usually considered as those with an average probability per flight hour of the order of 1 × 10^−9^, depending on the specific systems. Quantitative probability terms are also set to hazardous failure conditions that must be extremely remote (average probability of 1 × 10^−7^ per flight hour), and major failure conditions must be no more frequent than remote (average probability of 1 × 10^−5^ per flight hour).

Safety assessment consists of three phases, moving from the single devices or subsystems to the whole aircraft, as cross-effects and interactions are studied, including the influence of software and human interfaces.

Safety aspects are once more correlated to the number of parts and components. The reliability of N components is 1/N of the reliability of a single component. Therefore, as the system increases in size, the need to increase the reliability of the single element becomes stringent. This applies to all subsystems so that the use of morphing technology imposes the development of safer elements by several magnitudes. This can be a critical aspect of the realization process. A simple statement regards the evolution of the aircraft market. It is something that was generated before the COVID emergency, but it is a shared opinion that, after a contraction of the demand, former trends will be restored. If we think to a factor 10× of growth along a certain historical period, to maintain the absolute accident occurrence at the same level, the mean failure probability of the aircraft component shall decrease by the same quantity, arriving to 1 × 10^−10^ and 1 × 10^−11^, i.e., figures that pose an extreme challenge to technology. The introduction of morphing systems and their complexity, together with their larger number of parts, would decrease those numbers even more.

Additionally, specific maintenance procedures shall be developed to identify any hazards and ensure the continued airworthiness of the morphing system. In this respect, particular attention should be given to the design aspects to be emphasized in the design process to ensure easy and safe access to the components for fault isolation, replacement, inspection, and lubrication. The implementation of an adequate maintenance control program may also contribute to ensuring the structural integrity of critical components. 

Safety aspects may be dealt with the classical instruments of tools and fault trees, therefore there is no need to change from the process point of view. On the other side, that process will be longer and more articulated. Introduction of new systems require novel fault analyses that, in turn, have to be combined with the other system functions. Since combinations exhibit a factorial dependence on that number, it follows that times, complexity, and computational risks arise enormously.

The summary of weaknesses and criticalities associated to operations and safety of a morphing system is synthetically reported in Table 4.

## 7. Perspectives

### 7.1. Technology

In a morphing kinematic structure, the structural skeleton, the actuators, and the transmission line are something that is separated only in the ideas. Indeed, they form a sole subsystem with the intrinsic capability of moving to attain different shapes and bearing external loads. A current evolution is to integrate the design process for both of those elements; this partially innovative approach can lead to a certain decrease in the system complexity and parts, which may be accompanied by a slight reduction in the number of parts.

EMA actuators have clear limitations for the use in safety-critical applications. Jamming phenomena often require the redundancy of the system which cannot be afforded in an already-crowded application, such as morphing. Of course, this solution would also involve as cabling and routing impact, with severe consequences on the weight. On the other side, the growing use of such a kind of devices, which can be preferred to hydraulic devices for weight, volume, and maintenance aspects, gives optimal perspectives for the future.

The drawbacks associated with sensor systems may be overcome by using distributed (fiber optics) or wireless networks, which allows envisaging dramatic reductions in the interfaces, as well as cabling and routing [32]. A matter of discussion concerns the operation onboard of such systems; in that case, it should be noted that a number of onboard instruments have recently been produced by several firms concerning fiber Bragg gratings (FBG), and it is correct to think that these developments would continue to be more sophisticated devices. Wireless networks completely resolve the issue of cabling and routing, but there is also the problem of pointing out suitable transmission paths which shall face the consistency of data transmission onboard. Among the different possibilities, graphene-based techniques are worth being cited [33].

The impact of actuation systems, including the kinematic part (i.e., the link between generated forces and morphing structure), may find some relief in giving major attention to smart material-based devices. They would significantly affect the number of parts, allowing reductions in terms of volumes and weight. Of course, this would generate major needs for their development until certification; however, this aspect is mitigated if it is considered that more and more actuator systems of that kind have been developed and are currently mounted onboard of missiles and rockets, such as pin-pullers, exploiting shape memory alloys (SMA) technology. Such devices are finding more and more applications in several market sectors, including the automotive, heavy industry, as well as nuclear power plants. In the aeronautical field, some applications may be found on small UAV [34,35]. Recently, Boeing has installed an SMA torque tube within aircraft flight test hardware [36], while NASA tested SMA-actuated foldable wings in flight [37]. Moving from static to dynamic applications, piezoelectric materials may represent good alternatives to some common solutions, as shown on different class of rotorcraft [38,39].

As many times recalled, skins are perhaps the most critical element in a morphing system architecture. There are not many materials on the market which respond to the linked necessities, such as large strain absorption, low normal and high bending stiffness, durability, and possibly high damping. As large deformations are considered, fatigue issues may be relevant. Many solutions have been proposed, including hybrid metallic skins, auxetic materials, laminated composites, multi-stable composites, and so on [40,41,42,43]. Hybrid solutions, involving elastomeric materials and metallic parts, could give interesting answers in terms of protection from external agents and robustness from the structural point of view, while properly modulating rigidity properties.

### 7.2. Design

In spite of this, a kinematic morphing structure refers to assessed design tools and regulation requirements almost entirely, since standard components are generally referred to, and some points remain open. For instance, a number of configurations that should be tested to prove the reliability of the developed systems require specific attention by the stakeholders’ community, thus representing an issue not faced yet. It is believed that a smooth transition of morphing systems could be convenient, even from this point of view. A possible perspective could be represented by the use of adaptive devices as retrofit to existing aircraft; in this case, a lot of experience could be gained, exportable to many other items. The set-up of specifications would be easier, and this can be an important factor if it is considered that so much is still undefined when dealing with adaptive structures. Geometrical extension, shaping capability, and achievable benefits are still far from being consolidated features, especially given the lack of experience on the matter.

The assessment of dedicated tools is a matter on which some effort is expected in order to overcome the clear lack of numerical tools to deal with adaptive systems. Impressive steps were performed, as tools combining elastic behavior and rigid body motions appeared on the market. Now, those tools should be further expanded with the capability of considering large deformations (something different from large displacements), including the action of forces that are, in turn, a function of the achieved geometry, combined with detailed models of the singularity points (hinges, gears, and so on). In the same way, optimization tools which consider the different shapes seem to be necessary in order to achieve necessary weight reductions. It should be clear that such a numerical tool evolution is not just required for morphing systems, but it is something that should also be necessary for ordinary engineering. In fact, as the performance of the vehicle is targeted to increase, the necessity of a better simulation that can consider several neglected aspects becomes mandatory.

The usual way to deal with the presence of singularities, such as centers of rotation, is to neglect information concerning stress and strain values, provided by the numerical analysis (local values), and proceed with a classical semi-analytical tool for the design of the parts. This argument is something that has in itself great limitations, naturally converting into large safety factors and a de facto obstacle to allow the insertion of novel devices based on innovative or, even worse, revolutionary architectures.

These considerations hold even for aeroelastic simulations, where the behavior of the structure needs to be detailed at the most. The approach should include the many singularities that are in the loop, and allow for realizing multi-configuration optimization that can consider the whole geometrical domain where the adaptive structure operates. Combined with the former topic, a major challenge of aeroelasticity could be the capability of addressing model reductions that could move well beyond the usual standards. Indeed, pure numerical solutions are possible right now, which, however, can risk losing the physicality of the phenomenon. A suitable combination of enhanced methods, exploitation of the basic mathematics, and tools able to reverse the attained information into physical perspective may be a promising development path.

The most complex problem, which affects all the other themes introduced, concerns the coupling of the different systems. Such a complication is very hard to be considered in the design phase. Increasing the stiffness of the so-called fixed part of the aircraft would not improve the situation, leading to an unaffordable increase in weight. This kind of phenomenon may be dealt with through a complete re-thinking of the interface between adaptive and traditional parts, and the development of design tools able to consider the whole structural system from the beginning. Even in this case, the acquisition of a basic experience by the aircraft designers on the topics would certainly help the development process of these new tools.

### 7.3. Manufacturing and Assembly

The presence of a large number of parts is one of the most penalizing issues in the case of adaptive structural systems. Structural skeleton and actuation system may benefit new technologies by reducing the number of parts, namely additive layer manufacturing (ALM). In this way, it will be possible to reduce the number of parts, impressively overcoming a major drawback of kinematic morphing systems. This technique may also be conveniently applied to sensor systems with the ultimate perspective of printing the sensible network either, and may generally help to mitigate the complexity of the continuous transition of mechanical and physical features among materials of different origin and characteristics [44,45], including the use of smart materials [46]. Such a technology is already applied to UAV, contributing to merge different materials [47,48].

Currently, ALM has many showstoppers, moving from the cost of the implemented materials (many patented, and therefore their application is strongly restricted and expensive), and resistance characteristics of the products. On the other hand, it should be recognized that such a technology helps to realize shapes which are otherwise impossible, or extremely challenging, to realize by traditional processes. As already mentioned for morphing, even in this case it is necessary to evaluate the overall lifecycle cost after the implementation of ALM or traditional technology. In spite of that, however, additive layer manufacturing may be the right choice of certain morphing systems or some specific components of theirs. The latter are, in fact, characterized by a huge quantity of unique pieces that could be unaffordable to produce in series with the classical methods. As an example, wide actuator networks, deployed on a huge area of the wing, could have different needs of room and point layout, different for each location, in turn leading to the needs of parts of different size and even shape. Both gears of different size and shape could be easily realized by ALM machines, as any other part needing high values of tolerance and high precision, even avoiding the necessity of a massive number of pieces to warehouse. The model will be directly generated from CAD to the real world, passing through a common 3D printer. The assembly of these parts could be envisaged by expanded versions of the current machines, without using too much fantasy, but remaining in the ordinary evolution path. A gearbox can be easily thought of as produced directly by an augmented ALM printer. This step is essential to come to a proper reduction in the system complexity, managing, etc. Of course, specific steps, such as feasibility and reliability, should be assessed; however, again, this is part of the ordinary development track.

Whatever it is, the use of specific jigs and tools for properly assembling the different parts of an adaptive system is a necessary step. The very specialized applications currently require the design and realization of specific elements, the main objective of which is to properly handle deformable architectures without introducing unrecorded stress and strains that could activate unwanted permanent deformations, moving the system far from the design outline. Along this behavior, it is of interest to underline how, with investigating an adaptive system, possible deviation from the nominal shape (the shape “0”, above introduced) can be reset after assembly. As a drawback, such a reset occurs at expenses of the nominal excursions that should be then oversized to properly consider that possibility.

The integration of off-line subsystems may add further benefits. It is intended to separate the different parts for homogeneity and proceed separately with their integration. For instance, the sensor system may be integrated for each structural element that needs it onboard, opportunely designing component interfaces. The integration of actuation and structural systems has already been discussed. This strategy would be paid by a larger assembly time (a new step is introduced), but the assembly process would enjoy more simplicity and robustness.

A separate item deserves the skin manufacture. As of now, skin is seen as a separate part that is added to the structure after it has been realized. In that sense, it resembles a dress that should fit a certain body. Like a dress, it should be mounted and dismounted easily to host other similar elements. To close the similarity, it could be changed often like a dress, and its interaction with the operational environment is critical. It is clear that, if that is the current configuration, a reduction in costs, time of maintenance (also meaning cost and aircraft usability), and even weight shall be targeted in novel configurations. Hybrid solutions considering metals and elastomers are envisaged to be exploited for arrangements which are cheaper, lighter, and more robust.

### 7.4. Operations

ALM techniques may play an important role also from the point of view of maintenance and repair procedures. In fact, intervention and substitutions could be performed on-demand and on-site, significantly reducing the stock size.

Augmented reality (AR) could represent a valid answer to issues related to complex, unconventional systems, characterized by a high level of integration [49,50]. Such tools could be driven by criteria suited for the specific case, elaborate alternative strategies, and predict the achievable results and related risks. This approach, if supported by wide databases, for instance, by trained artificial neural networks (ANN), or any other kind of artificial intelligence (AI), could dramatically reduce operational times, allowing a selection of the most appropriate process without a direct implementation [51].

To enhance maintenance and repair processes, the use of health monitoring techniques combining structural and system features could be a winning solution. However, these systems, widely implemented in literature and in commercial applications, need some more evolutions for being implemented on aircraft, to a large extent. In the case of adaptive structural systems, the presence of large sensor networks could be exploited for monitoring the behavior of the different components, including structure, skins, and actuation architectures. Indeed, a fascinating solution could regard the monitoring of the sensor networks themselves by cross-observations, solving one of the problems correlated to largely distributed monitoring system. An additional interface with external systems could easily support and integrate the abovementioned AR tools [52].

Other developments that could support maintenance are represented by self-repair and damage prevention techniques. Self-repair capability may be implemented through the use of the so-called self-healing materials [53,54] which can recover some operational flaws to a certain extent. Highly integrated systems, even local, addressed to early detection of stress peaks may have a significant impact on maintenance, practically postponing any intervention.

Mainly, structure and actuation systems follow the usual repair paths, while something can be gained for sensors and skins. Non-reparable items suggest that they should be simply removed and substituted once failed. This can be only accomplished if interfaces are designed and implemented properly, with a sort of plug-and-play philosophy since sensored components shall maintain the capability of being fed and transmit information. As already mentioned, wireless systems could be the final development stage of the ongoing researches.

Safety issues are perhaps the most critical. The tremendous increase in components and parts can shift the cumulative safety level downwards. This fact, associated with a predicted increase in future air traffic (after the COVID emergency is over), could lead to unacceptable values. It is true that onboard systems are specifically devoted to move the aircraft wing and increase the magnitude. Predictions can move the expansion of air transportation by a similar factor in a one-generation period. In order to leave the number of incidents unchanged, the safety levels of the adopted components of an equal measure should be increased. In turn, and finally, this would mean to shift the reliability factor of the single component (probability of severe fault) from 1 × 10^−9^/1 × 10^−10^ to 1 × 10^−11^/1 × 10^−12^, which is probably impossible to reach within the current technology scenario development. Redundancy is a trivial way to solve that issue, but it is associated with tremendous impact, in terms of costs, weight, volume, and architecture complexity. Another possibility is to relegate structural adaptations to non-primary flight elements, but this choice leads to a strong reduction of the potential benefits of the morphing technology.

A statement should be devoted to the use of non-conventional elements. While smart actuators are an option, to be discussed and approved time by time, morphing skin implementation is a necessity. In such cases, safety aspects shall be dealt with very carefully. On one side, the material may not be known very well, giving rise in turn to excessive reliability factors that shall necessarily consider the lack of information. On the other side, their installation shall always be associated with non-critical parts or, equivalently, to a configuration whose collapse shall not cause catastrophic events. In any case, a reduction in interfaces may play a fundamental role in preserving the established safety levels without excessive penalty on the architecture of the reference system.

The summary of morphing technology perspectives, as analyzed in this chapter, is synthetically reported in Table 5.

## 8. Conclusions

In spite of some negativity that could arise from the analysis of the different components, a detailed survey of the posed problems and solutions shows that the perspectives are not far at all. In some cases, technology already exists, and has already been studied for a long time. In other cases, studies are already present in literature, even though they are still in their early stages. Finally, even if not so diffused, some needs have already been matched. For instance, morphing structures have already been manufactured, assembled, and successfully tested, even if to a limited extent.

In some sense, the aim of achieving the realization of adaptive operative aircraft may work as a pusher for attaining results otherwise expected in a longer period. The morphing idea can leverage technology, in a word. Conversely, morphing needs could also arise aspects otherwise neglected that could instead have a definitive role in the correct assessment of a specific technology. If integrated vehicle health monitoring is considered, for instance, the availability of structural monitoring, shape control, and kinematic observation systems may lead to an extreme intelligence of the structural system as a whole, allowing for outstanding data extraction and prediction.

There is also technology concurrence that could, in turn, foster adaptive structured development. Despite the ambition to reduce emissions, the full electric aircraft approach must face some important side effects, including energy storage of the batteries [55]; incompatibility of intrinsic weight with lightweight requirements [56]; and safety and certification, comprising power system failure, battery thermal runaway, and energy uncertainty [57]. Morphing technology could contribute by enhancing the performance of the aircraft, for instance, by increasing its operational range, strongly penalized by the limited performance of the batteries. The joined adoption of a morphing approach with laminar wing technology could lead to a net efficiency increase of about 20% [58].

Morphing implies a drastic reinterpretation of conventional design. In general, DOF continuous distribution leads to both the delocalization of its primary functions and the redundancy of them, which can lead to benefits in terms of aerodynamic efficiency, load distribution, and maneuverability [59]. The smoother shape of the deflected parts makes them more effective, with obvious benefits in terms of actuation power consumption [60]. Moreover, the high level of integration of subsystems favors clean architectures with a further improvement of performance. Finally, the above-mentioned design reinterpretation foresees an optimization of the wing structure, with a net saving of weight relating to conventional architectures, split into fixed and movable parts.

Moving to military, the so-called stealth combat aircraft may benefit morphing technology [61]. The smoothness of the surfaces, the absence of geometric discontinuities and asperities, together with the use of materials of new generation with a modest reflection profile in the electromagnetic spectrum, help to achieve a low observability. This justifies the large amount of theoretical and experimental investigations in this sense. An optimization process has been reported [62] which supports the design of morphing stealthy aerodynamic structures. Similar studies have been carried out for UAV [63]. The impact of morphing on stealth properties has been also addressed from an industrial point of view [64]. Examples of research activities on UCAV for implementing morphing technology to support and enhance stealth features are diffused, including CASC CH-7 UCAV and GJ-11 military drones in China [65], as well as B-2 bomber and monitoring drones in USA [66].

## Figures and Tables

**Table 1:** Summary of weakness and criticalities of a morphing system—components.

Components	Origin	Weakness	Impact
Structural Skeleton	DOF given	Number of parts Numb. of interfaces Number of joints	Complexity Weight
Sensor Network	DOF given	Cabling Number of devices	Complexity Weight Volume
Actuator Network	DOF given Room available Specifications	Number of devices Cabling Reliability Availability	Complexity Weight Volume Technology access
Control System	DOF given Requested functions	Stability	Complexity
Skin	Displacement targeted	Availability Fatigue resistance General deformability Type and number of joints	Shape degradation Weight Volume

**Table 2:** Summary of weakness and criticalities of a morphing system—design.

Step	Origin	Weakness	Impact
Approach	DOF given	Number of configurations	Time to assess Specifications issue
Tools	DOF given	Number of joints Lack of comprehensive tools Use of outdated methods	Deformability under loads Analysis of singularities Time to assess Simulation confidence Excessive conservativisms
Aeroelasticity	DOF given Geometry domain given	Number of configurations	Time to assess Balance complication Aeroelastic model size
Coupling	Number and location of morphing systems deployed	Interaction phenomena	Iterations Performance unpredictability Overall aircraft simulation

**Table 3:** Summary of weakness and criticalities of a morphing system—manufacturing and assembly.

Step	Origin	Weakness	Impact
Manufacture	Increased DOF Unconventional materials Conformal geometry	Many different parts Reduced size of many parts Needs of suited processes Needs of precision processes	Time of manufacturing Creation of specific tools Production chain enlargement Quality tests increase Tolerance reduction
Assembly	Increased DOF Pre-loaded components Load-bearing actuators Integrated sensors	Labile sub-structures Hard handling procedures Unconventional integration process interface increase	Dedicated jigs and tools Novel processes Evolution of standard processes Time of assembly

**Table 4:** Summary of weakness and criticalities of a morphing system—operations and safety.

Step	Origin	Weakness	Impact
Maintenance and Repair	Increased number of parts Integration of components Unconventional materials	Stock size Repair difficulty Increased intervals of inspections Lack of suited processes Behavior uncertainty	Time of intervention Increased costs Additional conservativisms Process restyling
Safety	Increased number of parts Increased number of subsystems Unconventional materials	Lack of suited processes Reliability factors approximation Increased interfaces	Complexity of analysis Additional conservativisms Process restyling

**Table 5:** Summary of morphing system technology perspectives.

Segment	Weakness	Mitigation approaches	Perspective
Technology	Number of parts Number of interfaces Number of joints Cabling Commercial availability Systems reliability	ALM implementation Bonding Wireless networks Multiplexed sensors Targeted technology development	Close Close Not so far Close Far away
Design	Configurations Number of joints Lack of representative tools Interaction phenomena	Augmented numerical tools Application on existing aircraft Augmented modelling of details	Not so far Current Close-
Manufacturing and Assembly	Number of parts Size of parts Number of interfaces Processes inadequate Labile sub-structures Skins	ALM implementation Process re-styling Design of specific jigs and tools Hybrid skin solutions	Close Not so far Current Not so far
Operations	Stock size Repair difficulty Increased inspection intervals Processes inadequate	ALM implementation HM implementation AR implementation Process re-styling	Close Close Current Not so far
Safety	Increased number of parts Increased number of subsystems Unconventional materials	Lack of suited processes Reliability factors approximation Increased interfaces	Complexity of analysis Additional conservativisms Process restyling

## Data Availability

No new data were created or analyzed in this study. Data sharing is not applicable to this article.

## References

[1] Flanagan J.S., Strutzenberg R.C., Myers R.B., Rodrian J.E. Development and flight testing of a morphing aircraft, the NextGen MFX-1. Proceedings of the 48th AIAA/ASME/ASCE/AHS/ASC Structures, Structural Dynamics, and Materials Conference.

[2] Aerospace, Spanwise Adaptive Wing (LEW-TOPS-124). Shape Memory Alloy Actuators Reconfigure Aircraft Wings in Flight. https://technology.nasa.gov/patent/LEW-TOPS-124.

[3] Jenett B.E., Calisch S., Cellucci D.W., Cramer N., Gershenfeld N., Swei S., Cheung K.C. (2017). Digital morphing wing: Active wing shaping concept using composite lattice-based cellular structures. Soft Robot..

[4] Cramer N.B., Cellucci D.W., Formoso O.B., Gregg C.E., Jenett B.E., Kim J.H., Lendraitis M., Swei S.S., Trinh G.T., Trinh K.V. (2019). Elastic shape morphing of ultralight structures by programmable assembly. Smart Mater. Struct..

[5] Cramer N.B., Tebyani M., Stone K., Cellucci D., Cheung K.C., Swei S., Teodorescu M. Design and testing of fervor: Flexible and reconfigurable voxel-based robot. Proceedings of the IEEE/RSJ International Conference on Intelligent Robots and Systems (IROS).

[6] Holle A.A. (1917). Plane and the like for Aeroplanes. U.S. Patent.

[7] Parker H.F. (1920). The Parker Variable Camber Wing.

[8] Hardy R. AFTI/F-111 mission adaptive wing technology demonstration program. Proceedings of the 1983 AIAA Aircraft Prototype and Technology Demonstrator Symposium, Air Force Museum.

[9] Bonnema K.L. (1988). AFTI/F-111 Mission Adaptive Wing Briefing to Industry. Air Force Wright Aeronautical Laboratories, Air Force Systems Command, Wright-Patterson Air Force Base.

[10] Hilbig R., Koerner H. Intelligente Tragfluegel—Aerodynamische Entwicklungsrichtungen fuer Verkehrsflugzeuge. Proceedings of the Deutsche Gesellschaft fuer Luft-und Raumfahrt (DGLR) Annual Convention.

[11] Woelcken P.C., Papadopoulos M. (2016). Smart Intelligent Aircraft Structures (SARISTU)—Proceedings of the Final Project Conference.

[12] NASA Tests Revolutionary Shape Changing Aircraft Flap for the First Time, Release 14-308, 7 November 2014. https://www.nasa.gov/press/2014/november/nasa-tests-revolutionary-shape-changing-aircraft-flap-for-the-first-time.

[13] Nguyen N.T., Ting E., Lebofsky S. Aeroelastic analysis of wind tunnel test data of a flexible wing with a variable camber continuous trailing edge flap (VCCTEF). Proceedings of the 56th AIAA/ASCE/AHS/ASC Structures, Structural Dynamics, and Materials Conference.

[14] Botez R.M., Koreanschi A., Sugar-Gabor O., Tondji Y., Guezguez M., Kammegne J.T., Grigorie L.T., Sandu D., Mebarki Y., Mamou M. (2018). Numerical and experimental transition results evaluation for a morphing wing and aileron system. Aeronaut. J..

[15] Meyran P., Pain H., Botez R.M., Laliberté J. (2021). Morphing winglet design for aerodynamic performance optimization of the CRJ-700 Aircraft, Part 1—Structural Design. INCAS Bull..

[16] Meyran P., Pain H., Botez R.M., Laliberté J. (2021). Morphing winglet design for aerodynamic performance optimization of the CRJ-700 Aircraft, Part 2—Control. INCAS Bull..

[17] Segui M., Abel F.R., Botez R.M., Ceruti A. (2021). New aerodynamic studies of an adaptive winglet application on the Regional Jet CRJ700. Biomimetics.

[18] Segui M., Mantilla M., Botez R.M. (2018). Design and validation of an aerodynamic model of the cessna citation x horizontal stabilizer using both OpenVSP and digital Datcom. Int. J. Mech. Ind..

[19] Segui M., Bezin S., Botez R.M. (2018). Cessna citation x performance improvement by an adaptive winglet during the cruise flight. Int. J. Mech. Ind..

[20] Noviello M.C., Dimino I., Concilio A., Amoroso F., Pecora R. (2019). Aeroelastic assessments and functional hazard analysis of a Regional aircraft equipped with morphing winglets. Aerospace.

[21] Sponsler G.C., Dare E., Gignoux N., Rubin N.N. (1973). The F-4 and the F-14, Columbia Research Corporation, Naval Analysis Programs—Office of Naval Research Grant N00014-72-C-0339.

[22] Goff W.E. (1971). Droop nose. Flight Int..

[23] Kudva J.N., Martin C.A., Scherer L.B., Jardine A.P., Rivas McGowan A.-M., Lake R.C., Sendeckyj G.P., Sanders B.P. Overview of the DARPA/AFRL/NASA smart wing program. Proceedings of the SPIE’s 6th Symposium on Smart Structures and Materials, Industrial and Commercial Applications of Smart Structures Technologies.

[24] Allison S.G., Fox R.L., Froggatt M.E., Childers B.A. THUNDER piezoelectric actuators as a method of stretch-tuning an optical fiber grating. Proceedings of the SPIE 7th Annual International Symposium on Smart Structures and Materials, Industrial and Commercial Applications of Smart Structures Technologies.

[25] Mehrpouya M., Gisario A., Broggiato G.B., Puopolo M., Vesco S., Barletta M. (2019). Effect of welding parameters on functionality of dissimilar laser-welded NiTi superelastic (SE) to shape memory effect (SME) wires. Int. J. Adv. Manuf. Technol..

[26] Drossel W.G., Junker T., Bucht A., y de Sosa I.N., Pagel K. (2016). Evaluation of shape memory alloy bulk actuators for wear compensation in ball screw drives. IFAC-PapersOnLine.

[27] Aroganam G., Manivannan N., Harrison D. (2019). Review on wearable technology sensors used in consumer sport applications. Sensors.

[28] Baitab D., Majid D.L.A.H.A., Abdullah E., Hamid M. (2018). A review of techniques for embedding shape memory alloy (SMA) wires in smart woven composites. Int. J. Eng. Technol..

[29] Materials Today Efficient and Unique—DuraAct Power Piezo Composite Patch Transducer with Multilayer Ceramics Uses d33 Effect. https://www.materialstoday.com/electronic-properties/products/duraact-power-piezo-composite-patch-transducer.

[30] IATA Airline Maintenance Cost Executive Commentary FY2019 Data. https://www.iata.org/contentassets/bf8ca67c8bcd4358b3d004b0d6d0916f/fy2019-mctg-report_public.pdf.

[31] Zheng M., Vu K.K., Liew J.Y.R. (2012). Aircraft morphing wing concepts with radical geometry change. IES J. Part A Civ. Struct. Eng..

[32] Ciminello M., Ameduri S., Romano F., Concilio A. (2020). Impact area and debonding line detection assessment by cross-correlation analysis and distributed sensing. Opt. Fiber Technol..

[33] Ameduri S., Ciminello M. Surface Bonding Graphene-Based Elastomeric Sensor: Preliminary Characterization of Adhesion Strength. Proceedings of the ASME 2019 Conference on Smart Materials, Adaptive Structures and Intelligent Systems.

[34] Pellone L., Ameduri S., Favaloro N., Concilio A. (2017). SMA-based system for environmental sensors released from an unmanned aerial vehicle. Aerospace.

[35] Ameduri S., Concilio A., Favaloro N., Pellone L. (2016). A shape memory alloy application for compact unmanned aerial vehicles. Aerospace.

[36] Xu L., Solomou A., Lagoudas D. A Three-Dimensional Constitutive Modeling for Shape Memory Alloys Considering Two-Way Shape Memory Effect and Transformation-Induced Plasticity. Proceedings of the AIAA Scitech 2019 Forum.

[37] Phys.org NASA Tests New Alloy to Fold Wings in Flight. https://phys.org/news/2018-01-nasa-alloy-wings-flight.html.

[38] Bashir M., Rajendran P. (2019). Recent trends in piezoelectric smart materials and its actuators for morphing aircraft development. IREME.

[39] Shivashankar P., Gopalakrishnan S. (2020). Review on the use of piezoelectric materials for active vibration, noise, and flow control. Smart Mater. Struct..

[40] Jani J.M., Leary M., Subic A., Gibson M.A. (2014). A review of shape memory alloy research, applications and opportunities. Mater. Des..

[41] Wang Z.P., Poh L.H., Zhu Y., Dirrenberger J., Forest S. (2019). Systematic design of tetra-petals auxetic structures with stiffness constraint. Mater. Des..

[42] Chillara V.S.C., Dapino M.J. (2020). Review of morphing laminated composites. Appl. Mech. Rev..

[43] Nicassio F., Scarselli G., Pinto F., Ciampa F., Iervolino O., Meo M. (2018). Low energy actuation technique of bistable composites for aircraft morphing. Aerosp. Sci. Technol..

[44] Ameduri S., Concilio A. (2020). Morphing wings review: Aims, challenges, and current open issues of a technology. Proc. Inst. Mech Eng. C J. Mech. Eng. Sci..

[45] Ajaj R.M., Beaverstock C.S., Friswell M.I. (2016). Morphing aircraft: The need for a new design philosophy. Aerosp. Sci. Technol..

[46] Prescouter The Top 6 Technologies for Improving Aircraft Fuel Efficiency. https://www.prescouter.com/2018/01/technologies-improving-aircraft-fuel-efficiency.

[47] Piedade A.P. (2019). 4D Printing: The shape-morphing in additive manufacturing. J. Funct. Biomater..

[48] Goh G.D., Agarwala S., Goh G.L., Dikshit V., Sing S.L., Yeong W.Y. (2017). Additive manufacturing in unmanned aerial vehicles (UAVs): Challenges and potential. Aerosp. Sci Technol..

[49] Intelligent Aerospace New Jamco Augmented Reality Technology Creates Efficient Maintenance Service. https://www.intelligent-aerospace.com/commercial/article/14168824/augmented-reality-aircraft-maintenance.

[50] De Crescenzio F., Fantini M., Persiani F., Di Stefano L., Azzari P., Salti S. (2011). Augmented reality for aircraft maintenance training and operations. IEEE Comput. Graph. Appl..

[51] Paul S., Kapoor K., Jasani D., Dudhwewala R., Gowda V.B., Nair T.R. Application of Artificial Neural Networks in Aircraft Maintenance, Repair and Overhaul Solutions. Proceedings of the International Conference on Total Engineering, Analysis and Manufacturing Technologies.

[52] Jeong Y., Son S., Jeong E., Lee B. (2018). An Integrated self-diagnosis system for an autonomous vehicle based on an IoT gateway and deep learning. Appl. Sci..

[53] Das R., Melchior C., Karumbaiah K.M., Rana S., Fangueiro R. (2016). Self-healing composites for aerospace applications. Advanced Composite Materials for Aerospace Engineering Processing, Properties and Applications.

[54] Nnamchi P.S., Obayi C.S., Tasaltin N., Nnamchi P.S., Saud S. (2020). Self-Healing in Titanium Alloys: A Materials Science Perspective. Advanced Functional Materials.

[55] The Conversation Electric Planes Are Here–but They Won’t Solve Flying’s CO₂ Problem. https://theconversation.com/electric-planes-are-here-but-they-wont-solve-flyings-co-problem-125900.

[56] Smithsonian Magazine Why Aren’t There Electric Airplanes Yet? It Comes Down to Batteries. https://www.smithsonianmag.com/innovation/why-arent-there-electric-airplanes-yet-it-comes-down-batteries-180970909/.

[57] Courtin C., Hansman R.J. Safety Considerations in Emerging Electric Aircraft Architectures. Proceedings of the 18th AIAA Aviation Technology, Integration, and Operations Conference.

[58] Aerospace Testing International Airbus Tests Laminar Flow Wing. https://www.aerospacetestinginternational.com/videos/airbus-tests-laminar-flow-wing.html.

[59] Ajaj R., Keane A., Beaverstock C., Friswell M., Inman D. Morphing Aircraft: The Need for a New Design Philosophy. Proceedings of the 7th Ankara International Aerospace Conference.

[60] Dimino I., Amendola G., Di Giampaolo B., Iannaccone G., Lerro A. Preliminary design of an actuation system for a morphing winglet. Proceedings of the 8th International Conference on Mechanical and Aerospace Engineering (ICMAE).

[61] The Warzone The Future Of Stealth Is “Morphing Wing” Technology, But Will The B-21 Feature It?. https://www.thedrive.com/the-war-zone/15413/the-future-of-stealth-is-in-morphing-wing-technology-will-the-b-21-feature-it.

[62] Li M., Bai J., Li L., Meng X., Liu Q., Chen B. (2019). A gradient-based aero-stealth optimization design method for flying wing aircraft. Aerosp. Sci. Technol..

[63] Hassanalian M., Quintana A., Abdelkefi A. (2018). Morphing and growing micro unmanned air vehicle: Sizing process and stability. Aerosp. Sci. Technol..

[64] Apuleo G., Concilio A., Dimino I., Lecce L., Pecora R. (2017). Aircraft Morphing—An Industry Vision. Morphing Wing Technologies Large Commercial Aircraft and Helicopters Scenario.

[65] The Warzone China Showcases Stealthier Sharp Sword Unmanned Combat Air Vehicle Configuration. https://www.thedrive.com/the-war-zone/30111/china-showcases-stealthier-sharp-sword-unmanned-combat-air-vehicle-configuration.

[66] Aerospace in Canada NASA and MIT’s “Morphing Wing” Concept. https://aerospaceincanada.com/index.php/2019/04/26/nasa-and-mits-morphing-wing-concept.

